# Neuritin inhibits astrogliosis to ameliorate diabetic cognitive dysfunction

**DOI:** 10.1530/JME-20-0321

**Published:** 2021-03-16

**Authors:** Zuo Zhang, Hongli Zhou, Jiyin Zhou

**Affiliations:** 1 National Drug Clinical Trial Institution, Second Affiliated Hospital, Army Medical University, Chongqing, China

**Keywords:** neuritin, diabetic cognitive dysfunction, gliosis, astrocyte, JAK2/STAT3 signaling pathway

## Abstract

Earlier, it was shown that reversing the downregulation of neuritin expression in the brain improves central neuropathy in diabetic rats. We investigated the protective mechanism of neuritin in diabetic cognitive dysfunction via astrocytes. Further, the impact of the overexpression of neuritin in the cortex and the hippocampus on diabetic cognitive dysfunction and astrogliosis in type 2 diabetic (db/db) mice was assessed. Antagonists were used to inhibit the JAK2/STAT3 signaling pathway in U-118MG, an astrocyte cell line. Immunofluorescence, Western blotting, and real-time PCR were performed. Neuritin overexpression in the hippocampus of db/db mice significantly ameliorated cognitive dysfunction, hippocampal neuronal impairment, and synaptic plasticity deterioration, and inhibited astrogliosis and the JAK2/STAT3 signaling pathway in the hippocampus. Neuritin suppressed the JAK2/STAT3 signaling pathway to inhibit lipopolysaccharide-induced gliosis in U-118MG cells. It was observed that neuritin regulates the JAK2/STAT3 signaling pathway in astrocytes to inhibit astrogliosis and improve diabetic cognitive dysfunction.

## Introduction

The incidence of cognitive dysfunction in patients with type 2 diabetes is 1.5 times higher than that in nondiabetic patients and 60 to 70% of diabetes patients have cognitive dysfunction. Currently, the management strategies for type 2 diabetes cannot benefit cognitive dysfunction, which place a great burden on type 2 diabetes, their families, and society (Yuan & Wang 2017). Progressive cognitive dysfunction is a central characteristic of diabetic encephalopathy (Xu *et al.* 2017). Prediabetes is linked to structural brain abnormalities, with further exacerbation of type 2 diabetes (van Agtmaal *et al.* 2018). Impaired cognition during type 2 diabetes is particularly evident in the scope of memory and executive function (Areosa Sastre *et al.* 2017).

Hippocampal synaptic plasticity is the neurobiological basis of learning and memory in cognitive function and participates in the occurrence and development of cognitive dysfunction in type 2 diabetes (Huang *et al.* 2016). Hippocampal synaptic plasticity is regulated by several neurotrophic factors, including neuritin (An *et al.* 2014). Astrocytes not only bridge the gap between metabolic supplies by blood vessels and neurons but also allow the fine control of neurotransmission by providing appropriate signaling molecules and insulation through tight enwrapping of synapses (Dallerac & Rouach 2016). Abnormalities in synaptic transmission lead to cognitive dysfunction (Koyama 2014). Astrocytes play an important role in cognitive functions, including learning and memory (Santello *et al.* 2019). Reactive gliosis is a process in which astrocytes maintain the stability of the neuronal microenvironment and play a protective role in the early stage of the injury, but persistent reactive gliosis forms a glial scar at the injury site to repair the missing site and further block the nerve. Diabetic rats display astrogliosis in the cortex and hippocampus (Tomassoni *et al.* 2013); while type 2 diabetic mice also show synaptic dysfunction and astrogliosis with memory impairment (Duarte *et al.* 2012).

Scar formation in astrocytes after spinal injury is regulated by the STAT3 signaling pathway (Wanner *et al.* 2013). The JAK2/STAT3 signaling pathway is involved in several diabetic complications, such as diabetic neuropathy (Li *et al.* 2019), diabetic cardiomyopathy (Gao *et al.* 2019), and diabetic nephropathy (Wang *et al.* 2012). The JAK2/STAT3 signaling pathway in the hippocampus of diabetic rats induced by streptozotocin is one of the most significant signaling pathways that regulates the process during such complications (Gurzov *et al.* 2016). The JAK2/STAT3 signaling pathway is involved in environmental contaminant-mediated astrocyte activation (Chen *et al.* 2018) and is necessary and sufficient to induce and maintain astrocyte reactivity (Ceyzeriat *et al.* 2018). Over the years, the JAK2/STAT3 signaling pathway has emerged as a central regulator of astrocyte reactivity and plays a critical role in animal models that regulate synaptic plasticity, reactive gliosis, and cognitive dysfunction (Ceyzeriat *et al.* 2016).

Neuritin (also named cpg15) is an activity-induced glycosylphosphatidylinositol-anchored axonal protein that is mainly expressed in the brain (Zhou & Zhou 2014). Neuritin ameliorates neurite outgrowth recovery of hippocampal neurons after mouse cerebral ischemia (Zhao *et al.* 2017) and improves depression and cognitive function during schizophrenia (Son *et al.* 2012). Nerve growth factor treatment restores neuritin levels in the dorsal root ganglia and sciatic nerves of diabetic rats (Karamoysoyli *et al.* 2008). In a previous study, we showed that berberine benefits diabetic neuropathy by improving micropathology and increasing neuritin expression via the mitogen-activated protein kinase signaling pathway (Zhou *et al.* 2016). Administration of exogenous neuritin improves the viability and function of Schwann cells in diabetic neuropathy rats (Xi *et al.* 2020).

In the present study, we employed the overexpression of neuritin in the cortex and hippocampus of type 2 diabetic (db/db) mice and lipopolysaccharide induction of U-118MG astrocyte cell line to investigate the effects of neuritin on diabetic cognitive dysfunction and astrogliosis through the JAK2/STAT3 signaling pathway.

## Material and methods

### db/db/neuritin/Emx1-Cre mice

A transgenic mouse line harboring the CMV-LoxP-STOP-LoxP-tagged human neuritin transgene was established using C57BL/6J mice generated by Cyagen Biosciences Inc. ((Guangzhou, China) Certificate No. TGBS141013BA1). A transgenic mouse model with high cortical and hippocampal tissue-specific overexpression of neuritin was established by crossing neuritin transgenic mice with Emx1-Cre mice (B6.129S2Emx1*^tm1(cre^^)^Krj*/J, https://www.jax.org/strain/005628). C57BL/6J-Leprdb/+ heterozygous littermate (db/m) mice were purchased from the Jackson Laboratory (Stock Number: 000699). Nondiabetic db/m mice were used to crossbreed C57BL/6J-Leprdb/db diabetic (db/db) mice. The db/m mice were crossed with the neuritin-Cre transgenic mice to yield db/db/neuritin/Exm1-Cre (neuritin overexpression db/db) mice, which are triple transgenic diabetic mice overexpressing neuritin in the cortex and hippocampus. All mice were backcrossed onto the C57BL/6J background for ten generations. Cre-mediated excision of neuritin was assessed by PCR using genomic DNA derived from the tail. In all animal studies, male mice were used, and littermates served as controls. All mice were bred in a specific pathogen-free, temperature-and humidity-controlled environment with a 12 h light: 12 h darkness cycle and allowed free access to food and water. The animal experiments were approved by the Army Medical University according to the guidelines of the Institutional Animal Care and Use Committee.

### Drug treatment

Both standard and high-fat diets containing 45% fat were purchased from Mediscience Ltd., Nanjing, China. The high-fat diet contained 24.0% protein, 41.0% carbohydrate, and 24.0% fat. Six-week-old male mice were separated into four groups, with six animals per group. One group of mice was fed a standard diet, while the other groups were fed a high-fat diet instead of a standard diet for 8 weeks. (i) db/m mice were fed a standard diet, (ii) db/db mice fed a high-fat diet, (iii) neuritin-overexpressing db/db mice fed a high-fat diet, (iv) db/db mice fed a high-fat diet + JAK2 inhibitor (AG490, 15 mg/kg, Abcam, Catalog #ab120950). During the 8 weeks of treatment, the mice were intraperitoneally injected with JAK2 inhibitor or its dilution vehicle (PBS containing 5% dimethyl sulfoxide) (Ignarro *et al.* 2013).

### Morris water maze test

After 7 weeks of drug administration, the animals were tested in a spatial version of the Morris water maze test as previously described (Si *et al.* 2016). The Morris water maze consisted of a circular water tank (120 cm diameter, 50 cm height) that was partially filled with water (25°C). Milk powder was used to render the water opaque. The training started by acclimating the mouse to the task environment with 2 days of free-swimming in the pool with no platform. Each session lasted for 2 min. The pool was virtually divided into four equal quadrants, labeled as N-S-E-W. A platform (10 cm diameter) was placed in one of the four maze quadrants (the target quadrant) and submerged 0.5 cm below the water surface. The platform remained in the same quadrant throughout the experiment. The mice were required to find the platform using only the distal spatial cues available in the testing room. The cues were maintained throughout the time of the test. The mice received four consecutive daily training trials in the following 5 days, with each trial having a ceiling time of 60 s and a trial interval of approximately 30 s. The mouse had to swim until it climbed onto the platform and then submerged beneath the water. After climbing onto the platform, the animal remained there for 30 s before the commencement of the next trial. The escape platform was kept at the same position relative to the distal cues. If the mouse failed to reach the escape platform within the maximum allowed time of 60 s, it was gently placed on the platform and allowed to remain there for the same amount of time. The time taken to reach the platform (latency in seconds) was measured.

A probe trial was performed to assess the extent of memory consolidation. The time spent in the target quadrant indicates the degree of memory consolidation that occurs after learning. In the probe trial, the mouse was placed into the pool as in the training trial, except that the hidden platform was removed from the pool. The time of crossing the former platform quadrant and the total time of crossing all quadrants were recorded for 60 s.

### Tissue preparation

After the Morris water maze test, mice were allowed to recover for a day, then fasted overnight, and were anesthetized with chloral hydrate (ip, 400 mg/kg). Blood of mice from each group was collected from the heart, transferred immediately into microcentrifuge tubes, and allowed to clot to obtain the serum. It was then perfused with 0.9% sodium chloride solution containing 0.1% diethylpyrocarbonate at 25°C followed by 4% paraformaldehyde in 0.1 mol/L PBS. After removing from the skull, the brains were fixed in 4% paraformaldehyde overnight, dehydrated in a 30% sucrose solution for 3–5 days at 4°C. Serial coronal sections (25 μm thick) of the whole hippocampus were cut using a sliding microtome and stored at −20°C until used for Nissl and immunofluorescence staining. The same sequence number section of serial sagittal sections of the brain containing the hippocampus was used for each experiment.

### Nissl staining

The frozen sections were fixed with 70% ethanol for 30 s and rinsed in DEPC-treated water for 30 s. The sections were then stained with 1% toluidine blue dye for 10 min at room temperature. After washing in distilled water for 1 min, the sections were dehydrated in a gradient alcohol and mounted with neutral resins. Nissl substance was observed under a light microscope (Olympus) with live neurons being highlighted by blue staining (Su *et al.* 2017). ImageJ 1.50 (National Institutes of Health) was used to analyze the average gray value of images.

### *In vitro* U-118MG cells experiment

U-118MG cells were maintained in a humidified incubator with 5% CO_2_ and maintained at 37°C in Dulbecco’s modified Eagle’s medium (DMEM), supplemented with 10% fetal bovine serum. Recombinant human neuritin (Sigma Co. Ltd.), JAK2 inhibitor (AG490), and STAT3 inhibitor (Stattic, Abcam Catalog #ab120952) were administered 30 min before stimulation with 1 μg/mL lipopolysaccharide. After 48 h of treatment, cells were collected and lysed, and cell extracts were analyzed by real-time PCR and Western blotting.

### Immunofluorescence staining

Enzymatic retrieval was performed by incubating the sections in proteinase K for 10 min at 25°C. The sections were rinsed with PBS, permeabilized with 0.3% Triton X-100 in PBS for 30 min, blocked using blocking buffer (PBS containing 5% normal serum and 0.3% Triton X-100) for 1 h, and incubated with primary antibodies (4°C, 12 h) and secondary antibodies (37°C, 2 h) in PBS containing 0.05% Tween 20. The primary antibodies used were as follows: GFAP (1:100, Abcam, Catalog #ab7260), JAK2 (1:100, Abcam, Catalog #ab108596), p-JAK2 (1:100, Abcam, Catalog #ab32101), STAT3 (1:100, Abcam, Catalog #ab68153), p-STAT3 (1:100, Abcam, Catalog #ab76315), and neuritin (1:100, Abcam, Catalog #64186). The secondary antibodies Alexa 488-labeled goat anti-rabbit IgG (1:500, Catalog#A0423) and Alexa 647-labeled goat anti-rabbit IgG (1:500, Catalog#A0468) were purchased from Beyotime (Shanghai, China). Nuclei were stained with DAPI (Beyotime). Finally, slides were washed five times in PBS and coverslips were mounted in 90% glycerol for microscopic analysis.

### Western blot

The cortex and hippocampus of the mice were dissected on ice. The proteins in the cortex, hippocampus, and U-118MG cells were extracted using RIPA lysis buffer (Beyotime, Catalog #P0013B), and total proteins in the supernatant were determined using a BCA protein assay kit (Beyotime, Catalog #P0012). Then, 40 μg of protein was mixed in a buffer (25% glycerol, 2% SDS, 0.01% bromophenol blue, Tris–HCl, pH 6.8) and heated at 100°C for 5 min. The samples were subjected to 10% SDS-PAGE, followed by transfer onto a PVDF membrane (Roche) using the GelDoc XR system (Bio-Rad) (Tang *et al.* 2017). The membrane was washed with Tris-buffered 154 mmol/L NaCl solution with 0.1% Tween 20, and incubated with anti-rabbit neuritin (Abcam, Catalog #64186), GFAP (Abcam, Catalog #ab7260), JAK2 (Abcam, Catalog # ab108596), p-JAK2 (Abcam, Catalog #ab32101), STAT3 (Abcam, Catalog #ab68153), p-STAT3 (Abcam, Catalog #ab76315), or β-actin polyclonal antibody (100 in dilution, Sigma, Catalog #A2103) for 1 h at 25°C, and incubated with peroxidase-conjugated anti-rabbit IgG (1:1000) for 1 h at 25°C. After the reaction, proteins were visualized with an ECL kit and images were obtained using ImageQuant LAS4010 (GE Healthcare). Samples were run in duplicate for each experiment. Densitometry analysis of the images was performed using ImageJ 1.50.

### Real-time PCR

Total RNA from the cortex, hippocampus, and U-118MG cells was extracted using RNAiso Plus (TAKARA, Catalog#9108/9109) (Tang *et al.* 2017). cDNA was synthesized using a Reverse-Transcription Reagent Kit (TAKARA, Catalog#RR047A) (Tang *et al.* 2017). Real-time PCR measurements of individual cDNAs were performed using SYBR *Premix Ex Taq*™ II (TAKARA, Catalog#RR820A) to measure the duplex DNA formation with the ABI Prism 7500 Sequence Detection System (Applied Biosystems) (Tang *et al.* 2017), normalized to the amount of β-actin RNA and analyzed by the 2^−∆∆CT^ method (Livak & Schmittgen 2001). The following primers were used: β-actin sense 5-CTCTAGACTTCGAGCAGGAGAT-3; β-actin antisense 5-CAGGATTCCATACCCAAGAAGG-3; neuritin sense 5-GCGGTGCAAATAGCTTACCTG-3, neuritin antisense 5-CGGTCTTGATGTTCGTCTTGTC-3′.

### Statistical analysis

All data are presented as means ± s.d. All grouped data were analyzed using SPSS 13.0. Comparisons between groups were made by one-way ANOVA followed by Tukey’s test to analyze the differences. Statistical significance was set at *P*  < 0.05.

## Result

### Overexpression of neuritin in hippocampus of mice

**Figure 1:**
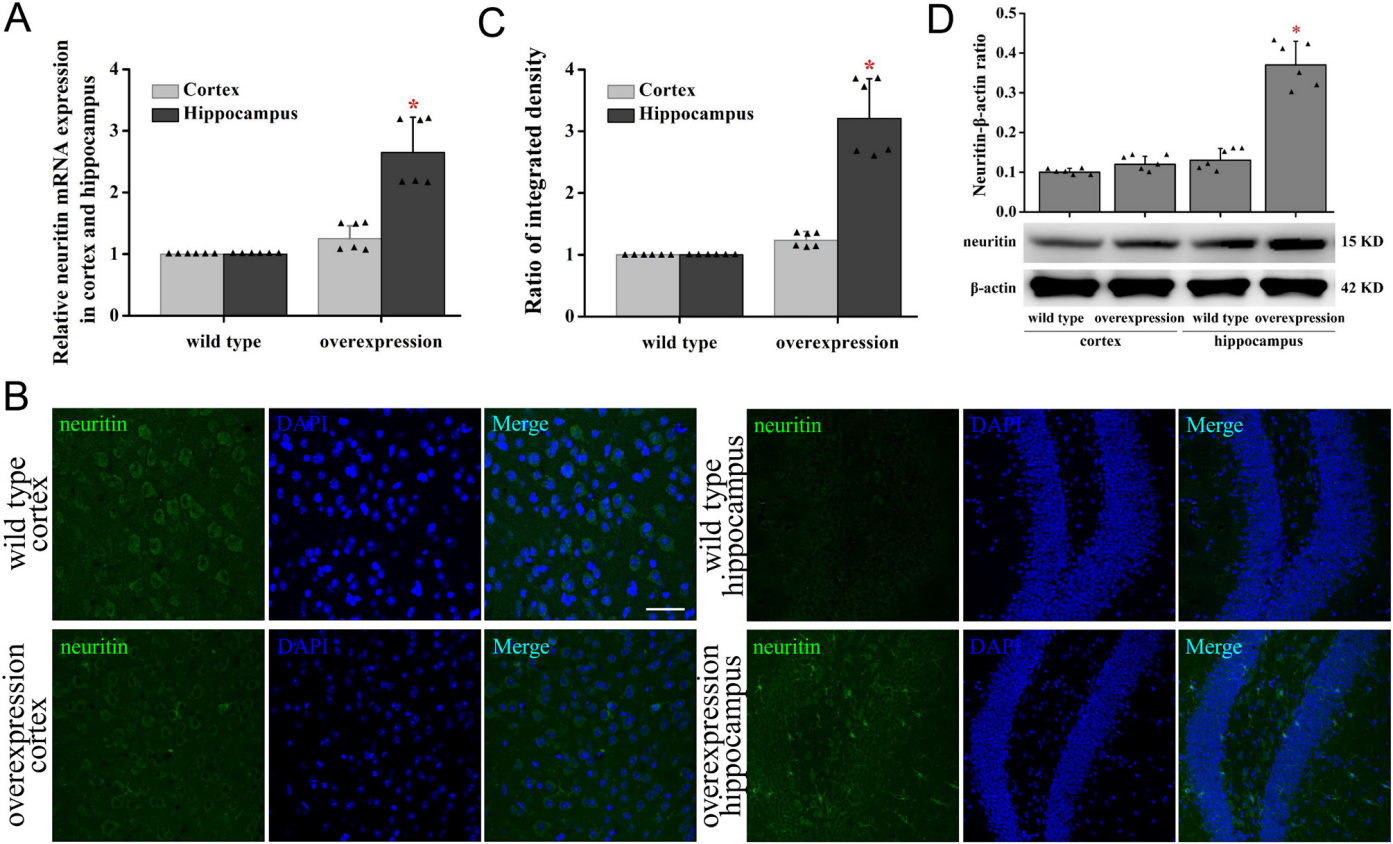
Expression profile of neuritin in the cortex and hippocampus of mice overexpressing neuritin. Neuritin mRNA expression in cortex and hippocampus was measured by real-time PCR in WT and neuritin-overexpressing mice (A). Neuritin expression in the cortex and hippocampus was observed by immunofluorescence (B), the quantification of fluorescence-integrated intensity (C), and by Western blot and its quantification (D) in WT and neuritin-overexpressing mice. Mean ± standard deviation (s.d.), *n* = 6. *P < 0.01, compared with WT mice. Overexpression, neuritin overexpression. A full color version of this figure is available at https://doi.org/10.1530/JOE-20-0321.

Real-time PCR analysis showed significantly increased mRNA expression of neuritin in the hippocampus of neuritin-overexpressing transgenic C57BL/6J mice compared to the WT mice but not in the cortex (Fig. 1A). Immunochemical staining (Fig. 1B) and Western blotting (Fig. 1C) analysis also confirmed increased neuritin expression. However, neuritin expression was not affected in the other tissues (data not shown). Since there was no difference in the expression of neuritin in the cortex, the subsequent experiments were only focused on the hippocampus.

### Effect of neuritin on memory

**Figure 2:**
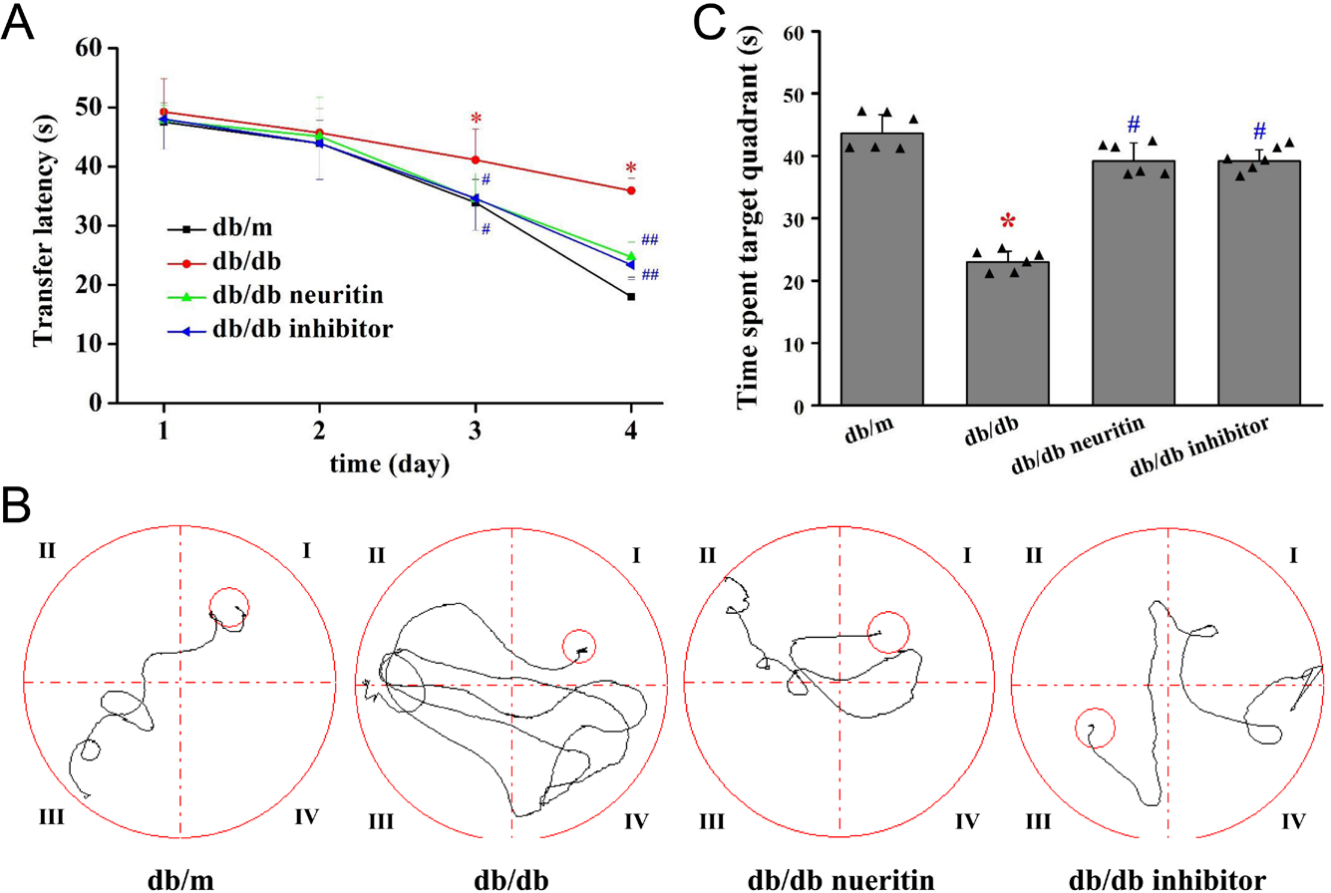
Effects of neuritin on cognitive dysfunction of db/db mice. The alteration of transfer latency (A), pathway maps of searching for the hidden platform at the fourth day of training (B), and the alteration of time spent in the target quadrant (C) during the Morris water maze test. Mean ± s.d., *n* = 6. *P < 0.01, compared with db/m mice; ^#^*P* < 0.01, compared with db/db mice. db/db neuritin, neuritin overexpression db/db; db/db inhibitor, db/db JAK2 inhibitor. A full color version of this figure is available at https://doi.org/10.1530/JOE-20-0321.

Cognitive function was assessed using the Morris water maze test. The mean escape latency for the trained mice decreased from 70 to 17 s over the course of the 20 learning trials. The mean escape latency did not differ between any of the groups on the first and the second days of testing in the Morris water maze. However, from the third day onwards, there was a significant difference in the transfer latency between db/db and db/m mice. db/db mice showed a lower ability to find the platform and learned its location on the fifth day of training. Neuritin overexpression significantly decreased the mean transfer latency in db/db mice (Fig. 2A). This poorer performance was also improved upon treatment with the JAK2 inhibitor, as evident from the animal’s decreased latency to find the platform from the third day of training. Figure 2B displays the representative swimming paths of mice in the four groups on the fourth day of training.

Animals showed a significant difference in the probe trial of the Morris water maze study, which measured how well the animals had learned and consolidated the platform location during the 5 days of training (Fig. 2C). db/db mice spent less time in the target quadrant than control mice. The time spent in the target quadrant was significantly higher in mice with neuritin overexpression and JAK2 inhibitor-treated db/db mice than in db/m mice.

### Effects of neuritin on body weight and brain weight

**Figure 3:**
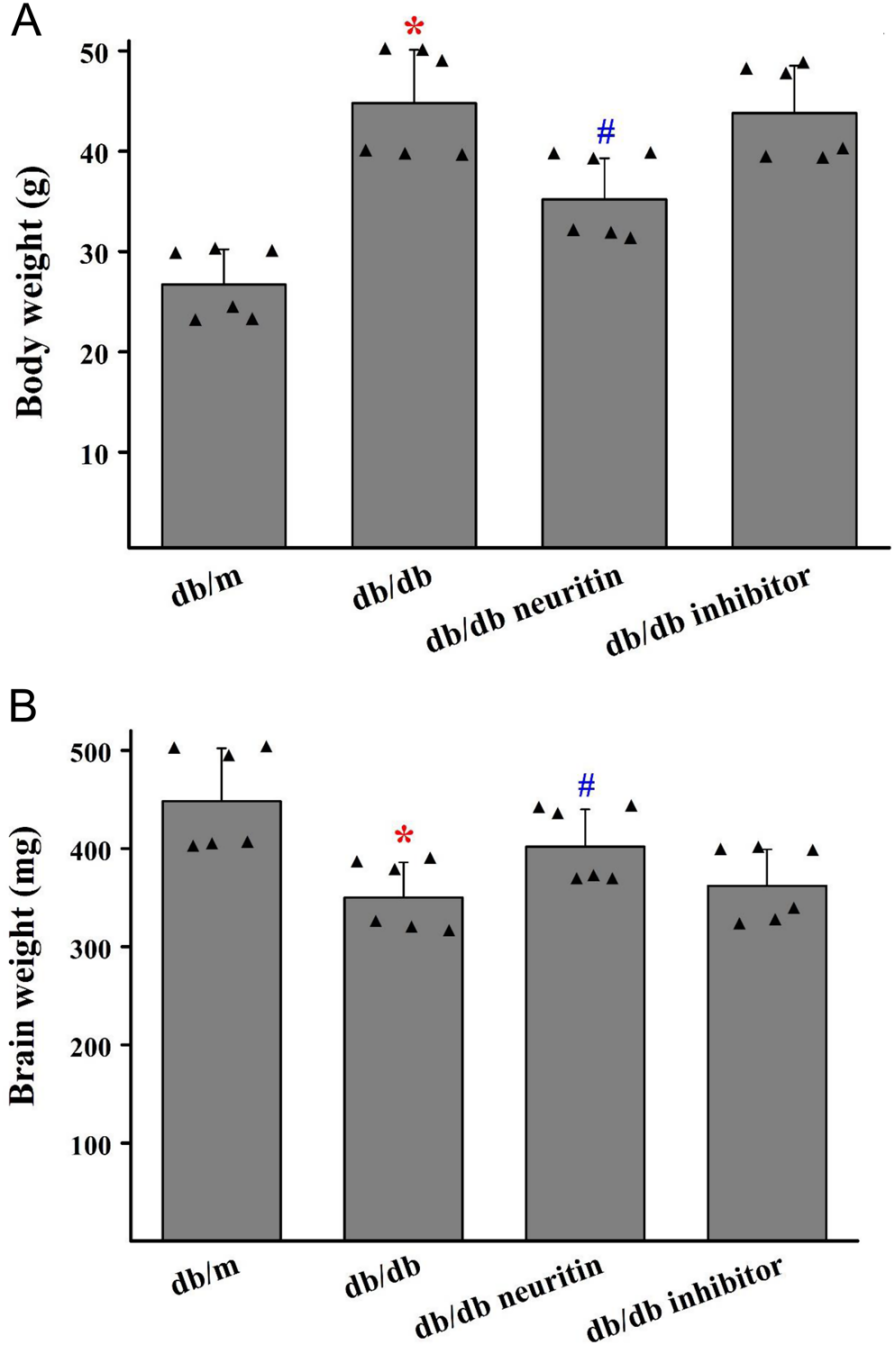
Effects of neuritin on body weight and brain weight of db/db mice. The changes in body weight (A) and brain weight (B). Mean ± s.d., *n* = 6. *P < 0.01, compared with db/m mice; ^#^*P* < 0.05, compared with db/db mice. db/db neuritin, neuritin overexpression db/db; db/db inhibitor, db/db JAK2 inhibitor. A full color version of this figure is available at https://doi.org/10.1530/JOE-20-0321.

As shown in Fig. 3A, db/db mice showed significantly higher body weight than db/m animals that were fed a standard diet. Neuritin overexpression slightly decreased the body weight of db/db mice However, JAK2 inhibitor administration for 8 weeks did not change the body weight of db/db mice.

db/db mice showed a significant decrease in brain weight compared to db/m mice (Fig. 3B). Neuritin overexpression slightly ameliorated the brain weight of db/db mice However, JAK2 inhibitor administration for 8 weeks did not affect the brain weight in db/db mice.

### Neuritin improved neuronal impairment in the hippocampus

**Figure 4:**
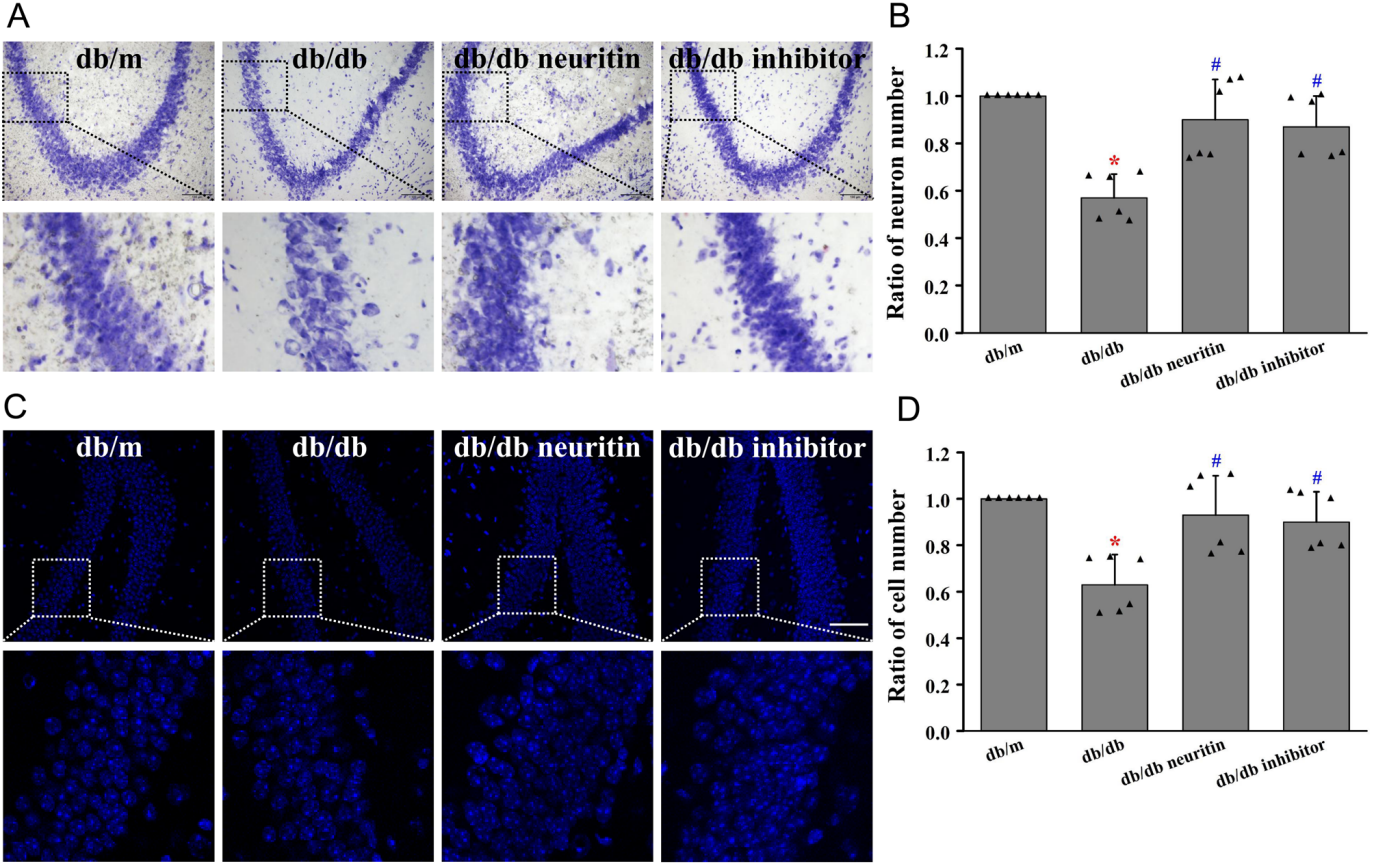
Neuritin ameliorated neuronal impairment in hippocampus. The Nissl staining (A) and its quantification analysis (B) and DAPI staining (C) and its quantification analysis (D) in the hippocampus. Mean ± s.d., *n* = 6. *P < 0.01, compared with db/m mice; ^#^ *P* < 0.01, compared with db/db mice. Scale bar = 100 μm in A, scale bar = 50 μm in B. db/db neuritin, neuritin overexpression db/db; db/db inhibitor, db/db JAK2 inhibitor. A full color version of this figure is available at https://doi.org/10.1530/JOE-20-0321.

Nissl staining revealed a significantly lower number of neurons in db/db mice than in db/m mice (Fig. 4A). DAPI staining showed a significantly lower number of all cells, including neurons, in db/db mice than in db/m mice (Fig. 4B). Overexpression of neuritin and JAK2 inhibitor treatment ameliorated these changes in the hippocampus of diabetic mice.

### Neuritin ameliorated astrogliosis and synaptic plasticity in hippocampus

**Figure 5:**
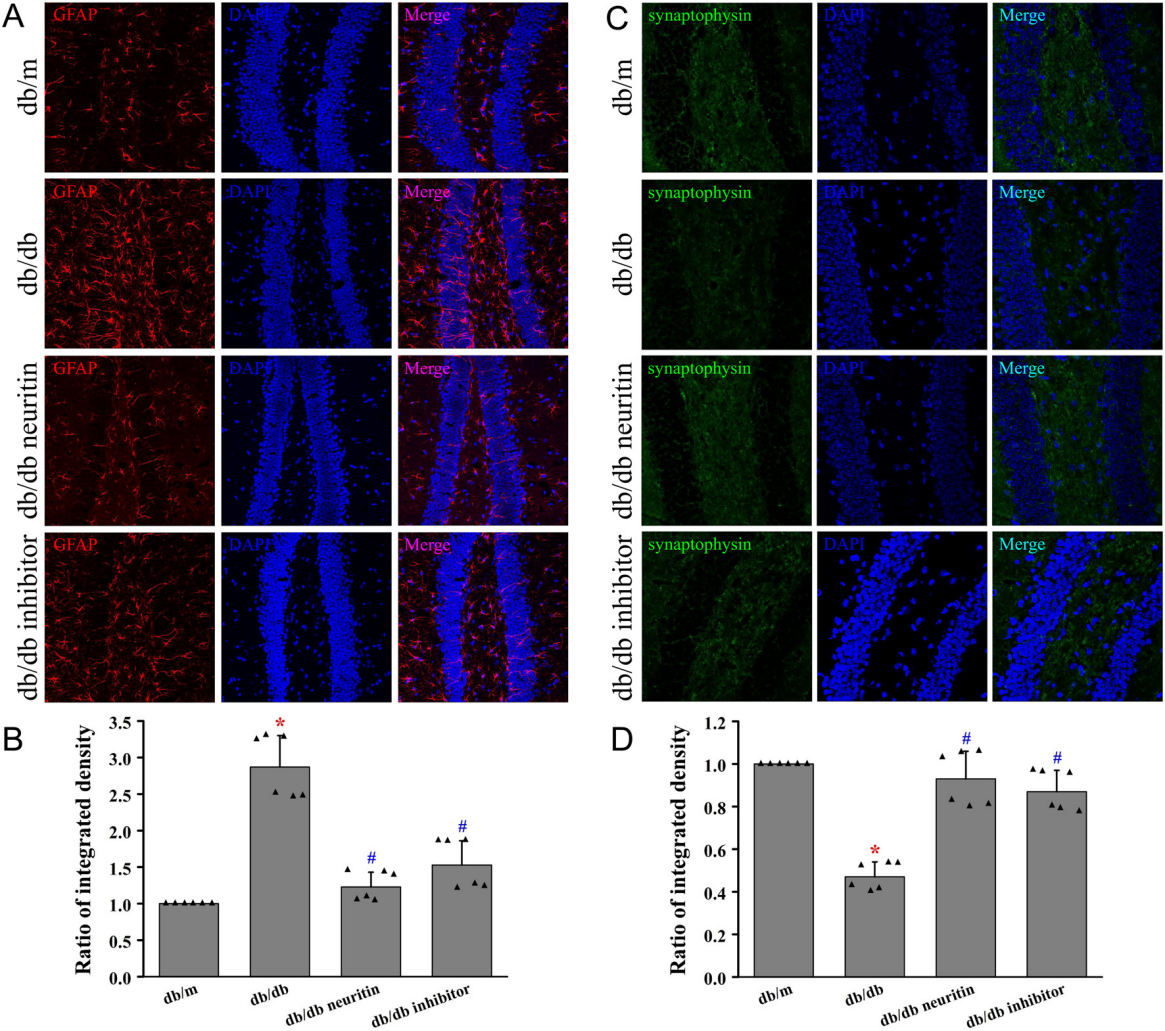
Neuritin ameliorated astrogliosis and synaptic plasticity in the hippocampus. GFAP protein was measured using immunofluorescence in the hippocampus (A) and its quantification of fluorescence integrated intensity (B). Synaptophysin protein was measured using immunofluorescence (C) and its quantification of fluorescence integrated intensity (D). Mean ± s.d., *n* = 6. *P < 0.01, compared with db/m mice; ^#^ *P* < 0.01, compared with db/db mice. db/db neuritin, neuritin overexpression db/db; db/db inhibitor, db/db JAK2 inhibitor. A full color version of this figure is available at https://doi.org/10.1530/JOE-20-0321.

In Fig. 5A, we demonstrated the astrocyte marker GFAP in the hippocampus of each group of mice by immunohistochemistry. There was a significant increase in the expression of GFAP in the hippocampus of db/db mice compared to that of standard diet-fed db/m mice. GFAP expression was downregulated by both neuritin overexpression and JAK2 inhibitor treatment in db/db mice.

db/db mice expressed lower levels of synaptophysin in the hippocampus than db/m mice (Fig. 5B). Overexpression of neuritin and JAK2 inhibitor treatment upregulated the expression of synaptophysin in the hippocampus of db/db mice.

### Neuritin regulated JAK2/STAT3 signaling pathway in hippocampus

**Figure 6:**
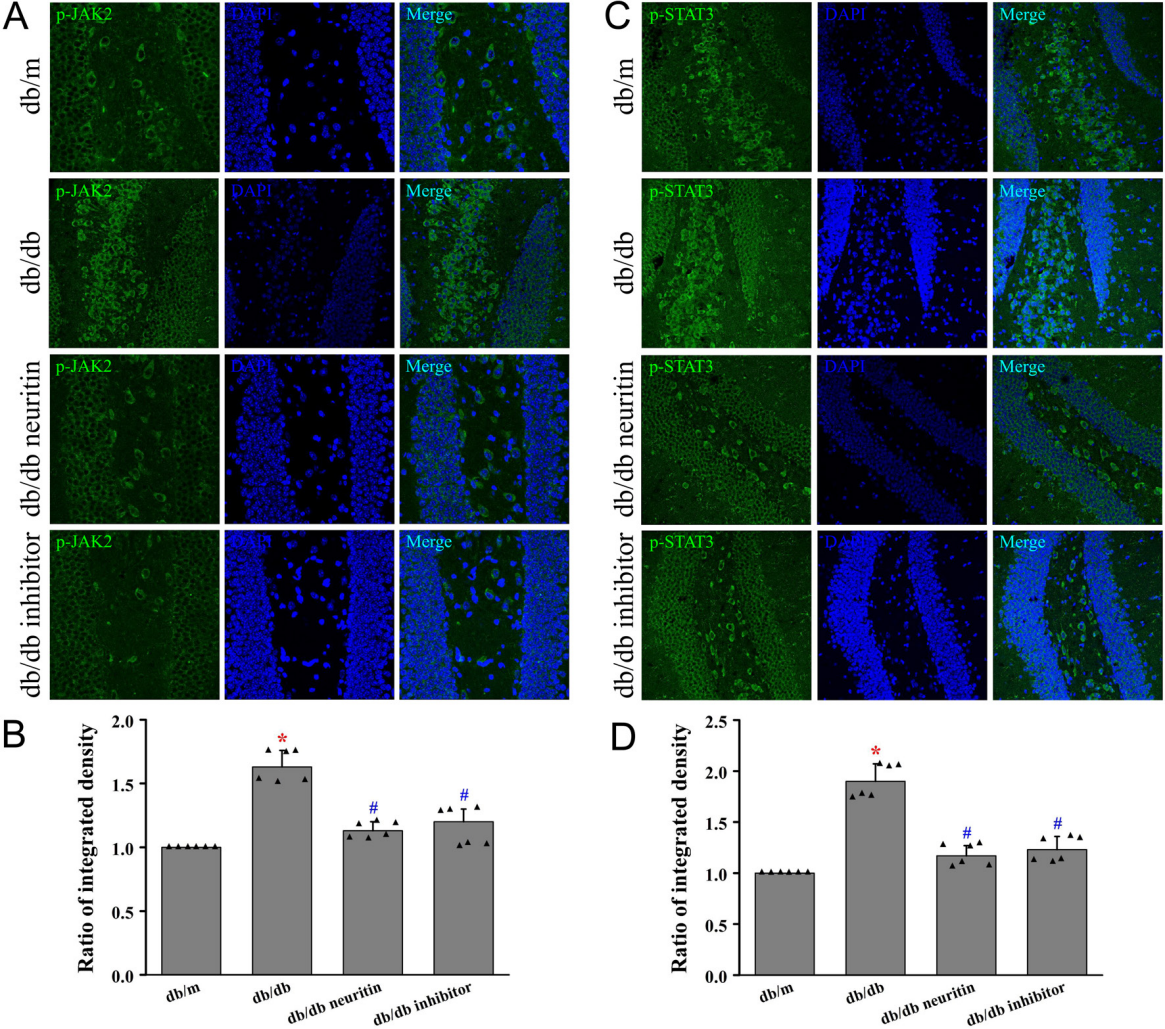
Neuritin suppressed JAK2/STAT3 signaling pathway in the hippocampus. Phosphorylation level of JAK2 was measured using immunofluorescence in hippocampus (A) and its quantification of fluorescence integrated intensity (B). Phosphorylation level of STAT3 was measured using immunofluorescence (C) and its quantification of fluorescence integrated intensity (D). Mean ± s.d., *n* = 6. *P < 0.01, compared with db/m mice; ^#^ *P* < 0.01, compared with db/db mice. db/db neuritin, neuritin overexpression db/db; db/db inhibitor, db/db JAK2 inhibitor. A full color version of this figure is available at https://doi.org/10.1530/JOE-20-0321.

To further explore the potential mechanism by which neuritin attenuates hippocampal astrogliosis, we determined the effect of neuritin on the JAK2/STAT3 signaling pathway. Protein expression of p-JAK2 and p-STAT3 was significantly upregulated in db/db mice compared to that in db/m mice, thereby indicating the activation of the JAK2/STAT3 signaling pathway (Fig. 6A and B). This activation was markedly reversed by the overexpression of neuritin and JAK2 inhibitor treatment, as shown by the significantly decreased expression of p-JAK2 and p-STAT3 in db/db mice. This provided compelling evidence that the neuritin interfered with the JAK2/STAT3 signaling pathway in the hippocampus.

### Neuritin inhibited lipopolysaccharide-induced gliosis in U-118MG cells

**Figure 7:**
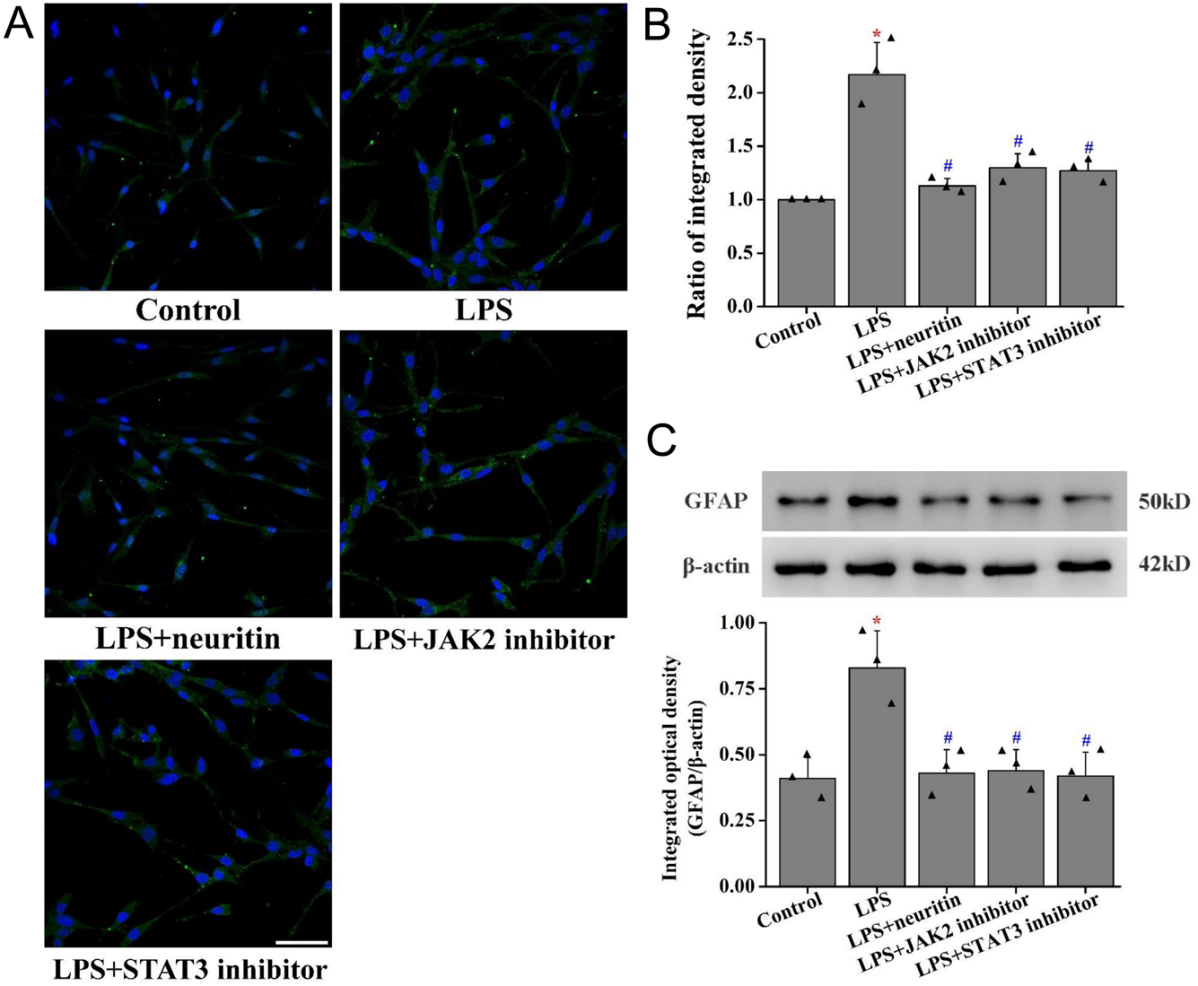
Neuritin inhibited gliosis in U-118MG cells. GFAP protein was measured using immunofluorescence in U-118MG cells (A) and its quantification of fluorescence-integrated intensity (B). GFAP protein was measured using Western blot and its quantification (C). Data are given as mean ± s.d. (*n* = 3). *P < 0.01 vs control cells; ^##^*P* < 0.01 vs lipopolysaccharide-induced cells. LPS, lipopolysaccharide. A full color version of this figure is available at https://doi.org/10.1530/JOE-20-0321.

Lipopolysaccharide significantly upregulated GFAP expression in U-118G cells compared to that in the control group (Fig. 7). However, when pretreated with recombinant neuritin (100 ng/mL), JAK2 inhibitor, or STAT3 inhibitor for 30 min, there was a suppressed GFAP expression.

### Neuritin suppressed lipopolysaccharide-stimulated JAK2/STAT3 pathway activation in U-118MG cells

**Figure 8:**
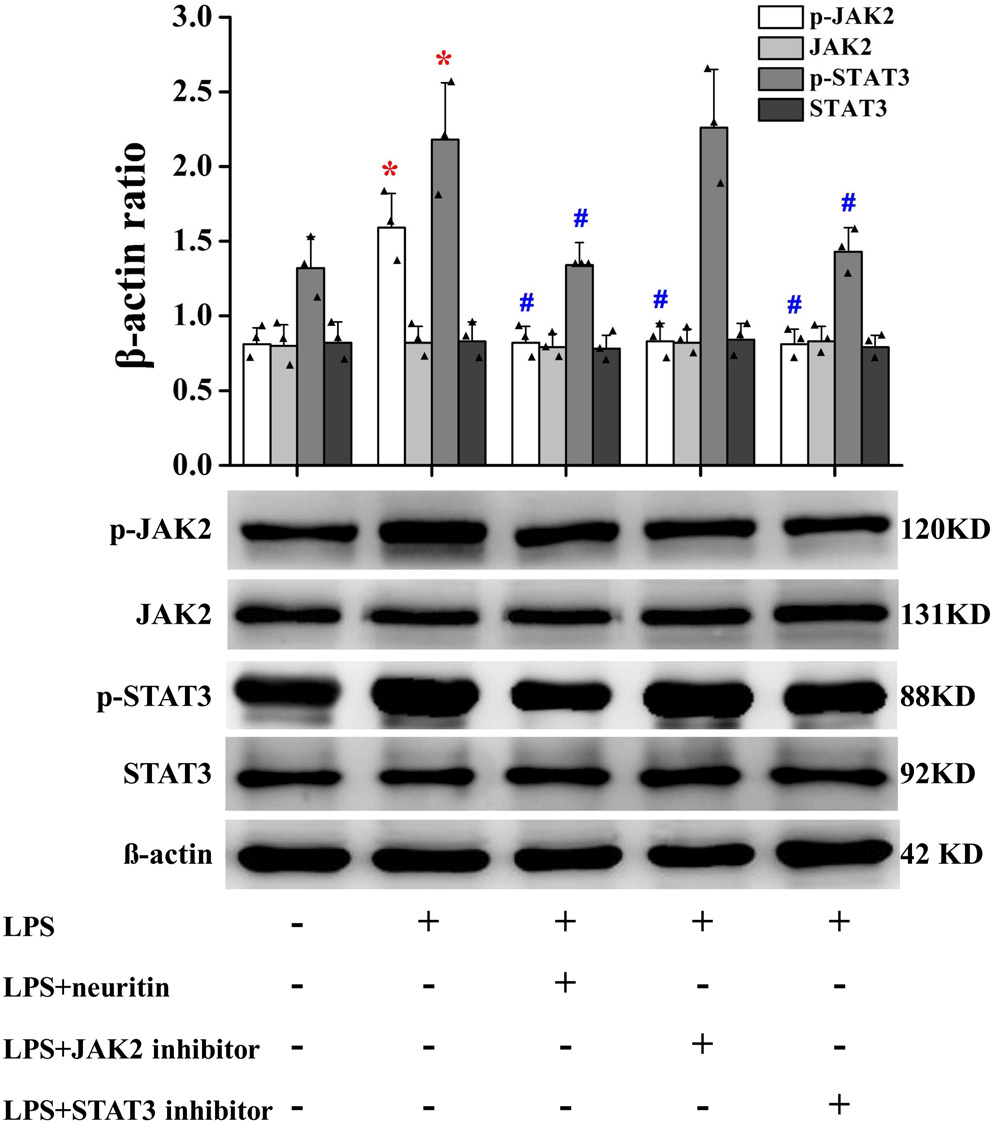
Neuritin inhibits JAK2/STAT3 signaling pathway in U-118MG cells. Data are given as mean ± s.d. (*n* = 3). *P < 0.01 vs control cells; ^##^*P* < 0.01 vs lipopolysaccharide-induced cells. LPS, lipopolysaccharide. A full color version of this figure is available at https://doi.org/10.1530/JOE-20-0321.

Lipopolysaccharide significantly increased the phosphorylation of JAK2 and STAT3 in U-118MG cells without affecting the total levels of the proteins. However, 30 min pretreatment with recombinant neuritin (100 ng/mL) decreased the phosphorylation of JAK2 and STAT3, while the phosphorylation of JAK2 and STAT3 showed a similar trend when the cells were incubated with JAK2 inhibitor. However, the STAT3 inhibitor only downregulated the expression of p-STAT3 (Fig. 8).

## Discussion

Our study found that neuritin overexpression in the hippocampus of db/db mice significantly ameliorated cognitive dysfunction, neuronal impairment, and synaptic plasticity, and inhibited astrogliosis and the JAK2/STAT3 signaling pathway in the hippocampus. Neuritin also suppressed the JAK2/STAT3 signaling pathway to inhibit lipopolysaccharide-induced gliosis in U-118MG cells.

Obesity is the single best predictor of whether a person would develop type 2 diabetes. In our study, db/db mice were fed a high-fat diet to induce diabetic neuropathy (Islam 2013). Our results show that there is significant downregulation in the expression of neuritin and increased body weight in db/db mice compared to control mice. The downregulated expression of neuritin might, thus, be body weight dependent, which is in accordance with the results of previous investigations in streptozotocin-induced diabetic rats (Karamoysoyli *et al.* 2008, Xi *et al.* 2020). There are no reports on the side effects of acute and chronic exogenous neuritin administration in mice or rats (An *et al.* 2014, Gao *et al.* 2016, Xi *et al.* 2020).

The diabetic brain has structural and functional abnormalities, including atrophy of the whole brain, gray matter, hippocampus, and amygdala. Progressive brain atrophy (Zhou *et al.* 2020), axon loss, and neuronal degeneration in the cortex and hippocampus have been observed in diabetic animals and humans (Klein & Waxman 2003). Consistent with previous reports in db/db mice showing that the brains of db/db mice are smaller and lighter than those of control mice (Sheppard *et al.* 1985, Makar *et al.* 1995, Vannucci *et al.* 1997, Ahima *et al.* 1999), our results showed that the brain weight of db/db mice was remarkably lower than that of db/m mice. According to the Jackson Laboratory, recombination (overexpression of neuritin) occurs in approximately 88% neurons of the neocortex and hippocampus, and in the glia of the cerebral cortex. Cortical excitatory neurons and glia (radial glia, astrocytes, and oligodendrocytes), but not GABAergic neurons, are produced in the Emx1-expressing lineage (Gorski *et al.* 2002). Overexpression of neuritin in the cortex and hippocampus increases the lower brain weight of db/db mice, but JAK2 inhibitor has no effect on the brain weight of db/db mice. Consistent with previous reports following Nissl and DAPI staining (Yan *et al.* 2009, Zhang *et al.* 2018), db/db mice showed a lower number of neurons and all cells in the hippocampus than that in db/m mice. Overexpression of neuritin prevents the loss of neurons in db/db mice, but JAK2 inhibitors could not restore the lost neurons in db/db mice. Global knockout of neuritin in mice accelerates retinal ganglion cell loss and retinal degeneration following optic nerve injury (Azuchi *et al.* 2018), while adeno-associated virus-mediated overexpression of neuritin delays retinal ganglion cell apoptosis, regenerates injured axons, and maintains retinal ganglion cell function following optic nerve injury (Sharma *et al.* 2015). Neuritin has similar protective effects against sciatic nerve injury in rats (Wang *et al.* 2016).

Diabetic central neuropathy is a critical complication of diabetes and is characterized by cognitive dysfunction and neurochemical and structural impairments (Sima 2010). Patients with type 2 diabetes have a higher risk of cognitive dysfunction, with deficits in short-term memory and executive function. Older type 2 diabetic patients have several impaired cognitive domains, with the highest ratio of impaired psychomotor speed (McCrimmon *et al.* 2012). In accordance with other reports (Infante-Garcia *et al.* 2017, Yan *et al.* 2019, Yermakov *et al.* 2019), in the Morris water maze test, diabetic mice showed exacerbated cognitive performance. Overexpression of neuritin ameliorated cognitive impairment, and the JAK2 inhibitor showed the same effects in diabetic mice.

Regional brain injury is tightly associated with neurocognitive impairment, especially in the hippocampus, which is mainly responsible for learning and memory (Stranahan *et al.* 2008). Synaptic plasticity is important for function of the neurons as plastic alterations in synaptic strength seem to be implicated in learning and memory (Bliss & Collingridge 1993). Synaptophysin is ubiquitous at the synapse, and therefore, synaptophysin immunostaining could be used for the quantification of synapses (Calhoun *et al.* 1996). Consistent with previous reports that diabetic mice show lower expression of synaptophysin in the hippocampus than control mice (Li *et al.* 2012, King *et al.* 2013, Porter *et al.* 2013), our results show that db/db mice expressed lower levels of synaptophysin in the hippocampus than db/m mice. Overexpression of neuritin and JAK2 inhibitor treatment upregulated the expression of synaptophysin in the hippocampus of db/db mice. Neuritin causes synapse formation and plasticity, neuritogenesis, neurite outgrowth, and neurite arborization, which participate in the development and function of the CNS (Putz *et al.* 2005, Fujino *et al.* 2011, Shimada *et al.* 2016, Subramanian *et al.* 2019).

Reactive astrocytes are essential in acute tissue remodeling and wound healing processes, eventually changing to scar-forming astrocytes and to become a dense glial scar (Okada *et al.* 2018). GFAP is strongly expressed in both reactive and scar-forming astrocytes. In several neurological diseases, reactive astrogliosis is a response to activated astrocytes. In most conditions, reactive astrogliosis can be considered a defensive reaction to resist acute stress, reversing CNS homeostasis, and preventing tissue damage. Continuing reactive astrogliosis can be maladaptive, resulting in the suppression of neural plasticity and regenerative responses (Pekny & Pekna 2014).

Astrocytes are active participants in synaptic processing and are involved in local synaptic plasticity. Astrocytes actively participate in normal memory function and abnormal processes, resulting in cognitive dysfunction under pathological conditions (Santello *et al.* 2019). Astrocytes regulate neuronal excitability, synaptic plasticity, and activity, and play a critical role in cognitive functions, such as learning and memory. Astrocyte regulation is considered as the focal point of processing cellular substrates for information and memory formation (Dallerac & Rouach 2016). Gliosis induced by astrocyte activation causes hippocampal neuronal impairment, leading to cognitive impairment (Shentu *et al.* 2019). Our results show that db/db mice have higher astrogliosis in the hippocampus than db/m mice. Neuritin overexpression ameliorates astrogliosis in db/db mice. Astrocyte degeneration, reactive astrogliosis and dystrophy are observed in Alzheimer’s disease (Shentu *et al.* 2019). In addition, astrocyte loss occurs at later stages of some neurodegenerative diseases, which might indirectly change neuronal function and survival (Rossi *et al.* 2008, Rodriguez *et al.* 2009, Martorana *et al.* 2012). However, another study showed that glial scar tissue formed after spinal cord injury might support neuron regeneration (Anderson *et al.* 2016).

Neuritin is an extracellular, glycosylphosphoinositide-linked protein that can be secreted in a soluble form by various cells, including neurons and astrocytes (Naeve *et al.* 1997, Putz *et al.* 2005, Zhao *et al.* 2017). One recent study found that the soluble form of neuritin from astrocytes repairs the damaged hippocampal neurons caused by ischemia by adhering to the neuronal surface. The increased expression of neuritin in astrocytes stimulated by ischemia might be triggered by modulation of cAMP response element-binding protein phosphorylation, mitogen-activated protein kinases, and phosphatidylinositide 3-kinases signaling pathways (Zhao *et al.* 2017).

Our results showed that the overexpression of neuritin suppressed the activated JAK2/STAT3 signaling pathway in the hippocampus of db/db mice and neuritin suppressed JAK2/STAT3 signaling pathway to inhibit lipopolysaccharide-induced gliosis in U-118MG cells. Other studies have shown that astrogliosis has the potential to interfere with synapse sprouting (Cirillo *et al.* 2012, Shentu *et al.* 2019) and is associated with the JAK2/STAT3 signaling pathway (Robson *et al.* 2014). Astrogliosis might also damage neuronal survival, which is ameliorated by JAK2 inhibition (Ignarro *et al.* 2013). LPS induces the activation of retinal astrocytes via the JAK2/STAT3 signaling pathway (Jang *et al.* 2007). The JAK2/STAT3 signaling pathway also contributes to the development of diabetic macrovascular complications by mediating inflammation associated with vascular endothelial cells and/or monocytes (Yang *et al.* 2017) and involves the renal protective effect of paeoniflorin (Li *et al.* 2018).

In conclusion, neuritin overexpression in the hippocampus of db/db mice significantly ameliorated cognitive dysfunction, hippocampal neuronal impairment, and synaptic plasticity deterioration, and suppressed astrogliosis and the JAK2/STAT3 signaling pathway in the hippocampus. Neuritin regulates the JAK2/STAT3 signaling pathway to inhibit lipopolysaccharide-induced gliosis in U-118MG cells. Therefore, neuritin might at least partly regulate the JAK2/STAT3 signaling pathway to inhibit astrogliosis and improve diabetic cognitive dysfunction.

## Declaration of interest

The authors declare that there is no conflict of interest that could be perceived as prejudicing the impartiality of the research reported.

## Funding

This study is supported by grants from the National Natural Science Foundation of China (Nos. 81770806), the Chongqing Natural Science Foundation of China (No. CSTC2016shmszx130006), and Special Project for Enhancing Science and Technology Innovation Ability of Army Military Medical University (No. 2019XYY16).

## Author contribution statement

J Z designed the experiments; Z Z and H Z performed the experiments; Z Z and H Z analyzed the data; Z Z, H Z and J Z wrote the manuscript; Z Z, H Z and J Z edited the manuscript; Z Z, H Z and J Z read and approved the final manuscript.
